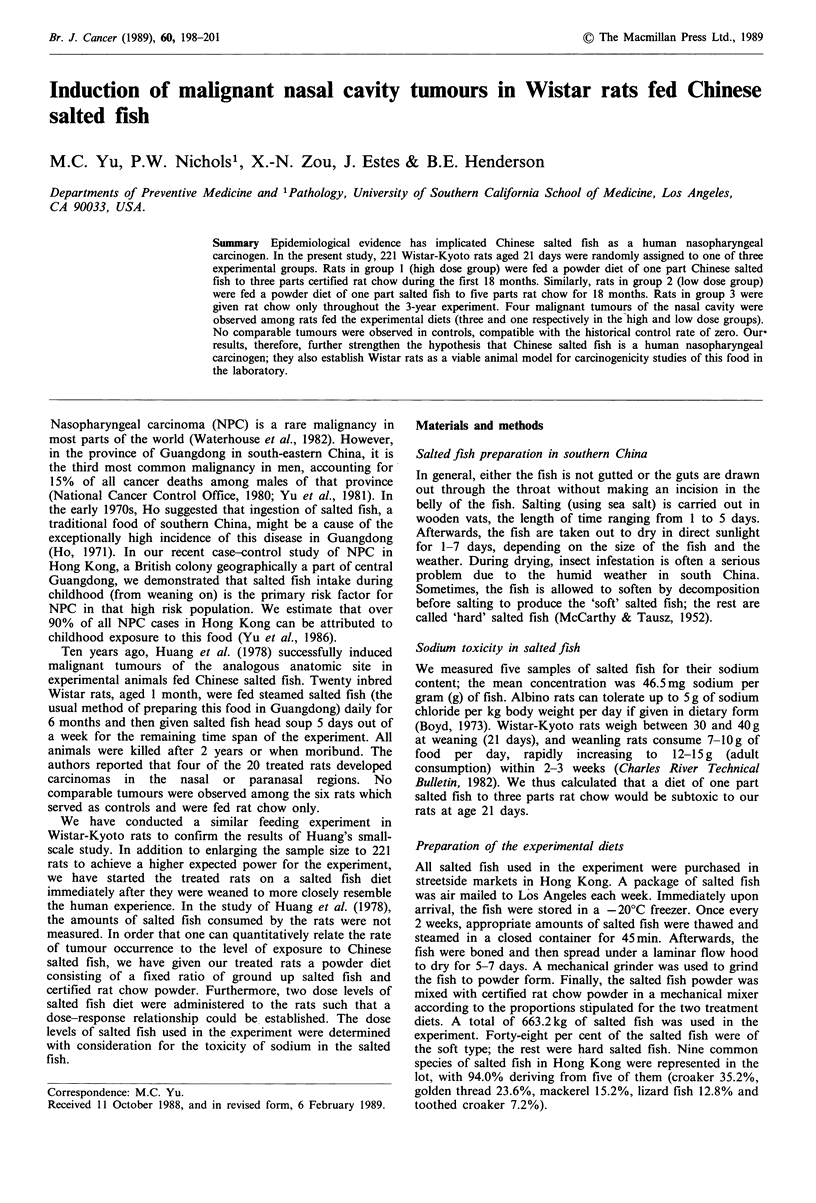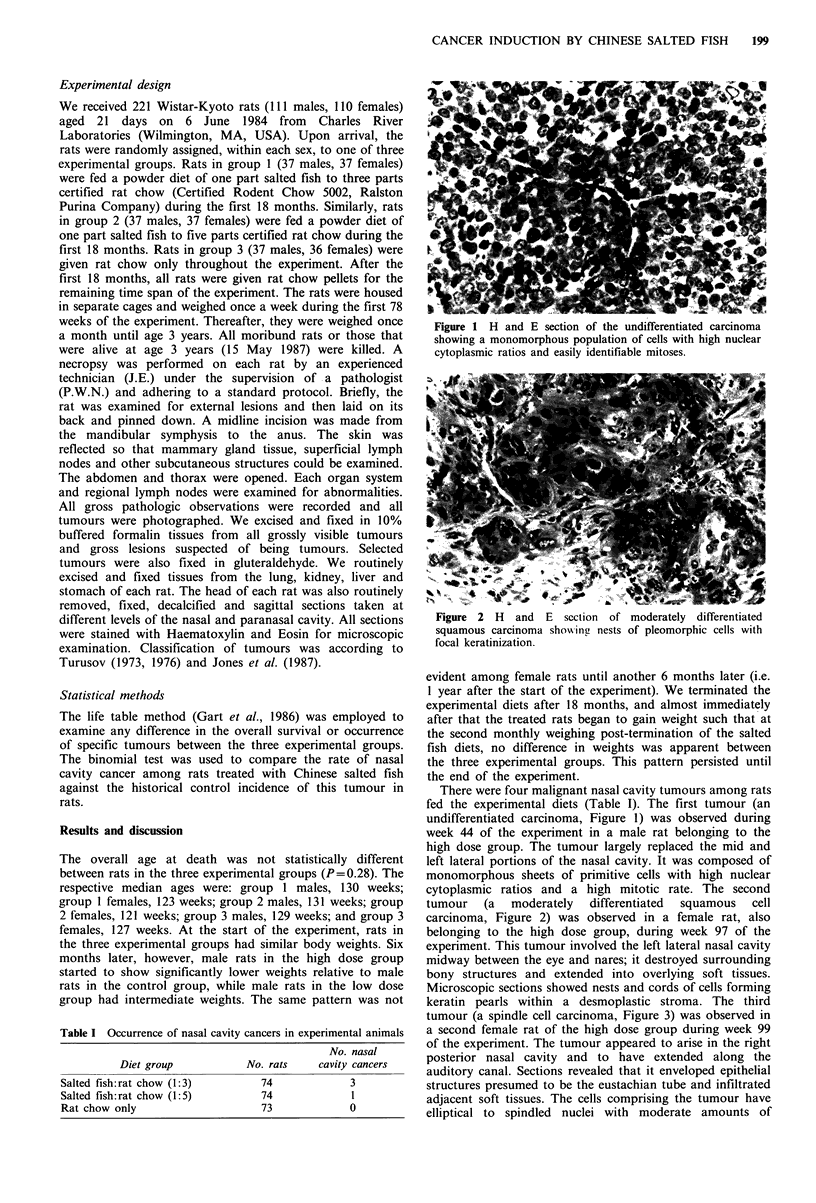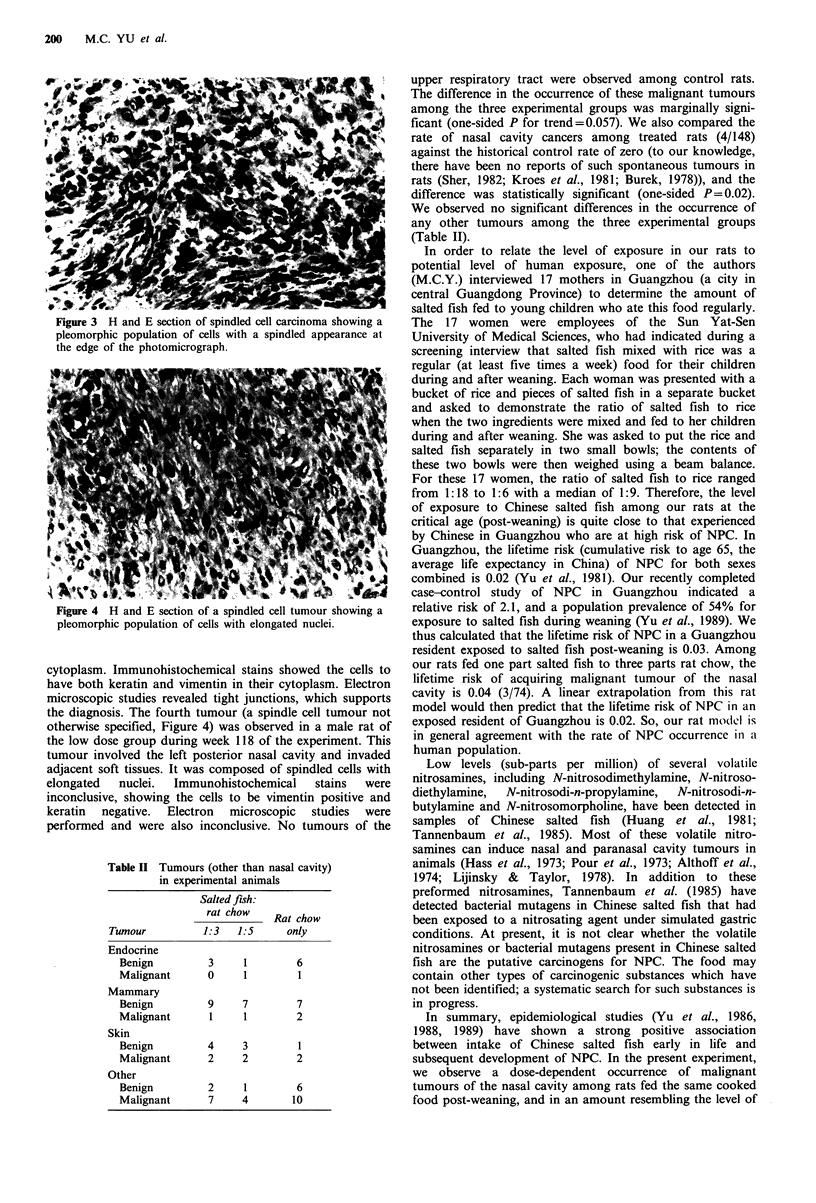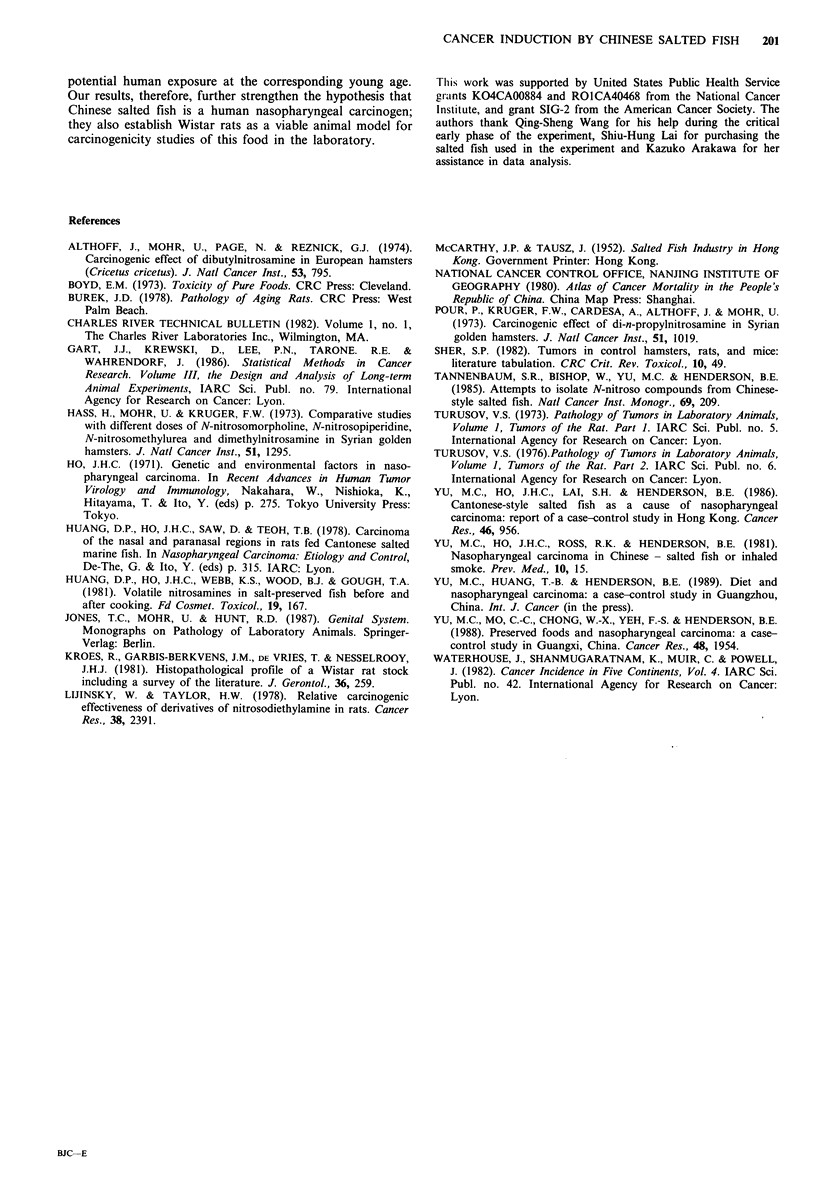# Induction of malignant nasal cavity tumours in Wistar rats fed Chinese salted fish.

**DOI:** 10.1038/bjc.1989.250

**Published:** 1989-08

**Authors:** M. C. Yu, P. W. Nichols, X. N. Zou, J. Estes, B. E. Henderson

**Affiliations:** Department of Preventive Medicine, University of Southern California School of Medicine, Los Angeles 90033.

## Abstract

**Images:**


*Br. J. Cancer* (1989), **60**, 198–201 © The Macmillan Press Ltd., 1989

### Induction of malignant nasal cavity tumours in Wistar rats fed Chinese salted fish

M.C. Yu, P.W. Nichols[1], X.-N. Zou, J. Estes & B.E. Henderson

*Departments of Preventive Medicine and [1]Pathology, University of Southern California School of Medicine, Los Angeles, CA 90033, USA.*

**Summary** Epidemiological evidence has implicated Chinese salted fish as a human nasopharyngeal carcinogen. In the present study, 221 Wistar-Kyoto rats aged 21 days were randomly assigned to one of three experimental groups. Rats in group 1 (high dose group) were fed a powder diet of one part Chinese salted fish to three parts certified rat chow during the first 18 months. Similarly, rats in group 2 (low dose group) were fed a powder diet of one part salted fish to five parts rat chow for 18 months. Rats in group 3 were given rat chow only throughout the 3-year experiment. Four malignant tumours of the nasal cavity were observed among rats fed the experimental diets (three and one respectively in the high and low dose groups). No comparable tumours were observed in controls, compatible with the historical control rate of zero. Our results, therefore, further strengthen the hypothesis that Chinese salted fish is a human nasopharyngeal carcinogen; they also establish Wistar rats as a viable animal model for carcinogenicity studies of this food in the laboratory.

Nasopharyngeal carcinoma (NPC) is a rare malignancy in most parts of the world (Waterhouse *et al.*, 1982). However, in the province of Guangdong in south-eastern China, it is the third most common malignancy in men, accounting for 15% of all cancer deaths among males of that province (National Cancer Control Office, 1980; Yu *et al.*, 1981). In the early 1970s, Ho suggested that ingestion of salted fish, a traditional food of southern China, might be a cause of the exceptionally high incidence of this disease in Guangdong (Ho, 1971). In our recent case–control study of NPC in Hong Kong, a British colony geographically a part of central Guangdong, we demonstrated that salted fish intake during childhood (from weaning on) is the primary risk factor for NPC in that high risk population. We estimate that over 90% of all NPC cases in Hong Kong can be attributed to childhood exposure to this food (Yu *et al.*, 1986).

Ten years ago, Huang *et al.* (1978) successfully induced malignant tumours of the analogous anatomic site in experimental animals fed Chinese salted fish. Twenty inbred Wistar rats, aged 1 month, were fed steamed salted fish (the usual method of preparing this food in Guangdong) daily for 6 months and then given salted fish head soup 5 days out of a week for the remaining time span of the experiment. All animals were killed after 2 years or when moribund. The authors reported that four of the 20 treated rats developed carcinomas in the nasal or paranasal regions. No comparable tumours were observed among the six rats which served as controls and were fed rat chow only.

We have conducted a similar feeding experiment in Wistar-Kyoto rats to confirm the results of Huang's small-scale study. In addition to enlarging the sample size to 221 rats to achieve a higher expected power for the experiment, we have started the treated rats on a salted fish diet immediately after they were weaned to more closely resemble the human experience. In the study of Huang *et al.* (1978), the amounts of salted fish consumed by the rats were not measured. In order that one can quantitatively relate the rate of tumour occurrence to the level of exposure to Chinese salted fish, we have given our treated rats a powder diet consisting of a fixed ratio of ground up salted fish and certified rat chow powder. Furthermore, two dose levels of salted fish diet were administered to the rats such that a dose–response relationship could be established. The dose levels of salted fish used in the experiment were determined with consideration for the toxicity of sodium in the salted fish.

Correspondence: M.C. Yu.
Received 11 October 1988, and in revised form, 6 February 1989.

### Materials and methods

### *Salted fish preparation in southern China*

In general, either the fish is not gutted or the guts are drawn out through the throat without making an incision in the belly of the fish. Salting (using sea salt) is carried out in wooden vats, the length of time ranging from 1 to 5 days. Afterwards, the fish are taken out to dry in direct sunlight for 1–7 days, depending on the size of the fish and the weather. During drying, insect infestation is often a serious problem due to the humid weather in south China. Sometimes, the fish is allowed to soften by decomposition before salting to produce the 'soft' salted fish; the rest are called 'hard' salted fish (McCarthy & Tausz, 1952).

### *Sodium toxicity in salted fish*

We measured five samples of salted fish for their sodium content; the mean concentration was 46.5 mg sodium per gram (g) of fish. Albino rats can tolerate up to 5 g of sodium chloride per kg body weight per day if given in dietary form (Boyd, 1973). Wistar-Kyoto rats weigh between 30 and 40 g at weaning (21 days), and weanling rats consume 7–10 g of food per day, rapidly increasing to 12–15 g (adult consumption) within 2–3 weeks (*Charles River Technical Bulletin*, 1982). We thus calculated that a diet of one part salted fish to three parts rat chow would be subtoxic to our rats at age 21 days.

### *Preparation of the experimental diets*

All salted fish used in the experiment were purchased in streetside markets in Hong Kong. A package of salted fish was air mailed to Los Angeles each week. Immediately upon arrival, the fish were stored in a −20°C freezer. Once every 2 weeks, appropriate amounts of salted fish were thawed and steamed in a closed container for 45 min. Afterwards, the fish were boned and then spread under a laminar flow hood to dry for 5–7 days. A mechanical grinder was used to grind the fish to powder form. Finally, the salted fish powder was mixed with certified rat chow powder in a mechanical mixer according to the proportions stipulated for the two treatment diets. A total of 663.2 kg of salted fish was used in the experiment. Forty-eight per cent of the salted fish were of the soft type; the rest were hard salted fish. Nine common species of salted fish in Hong Kong were represented in the lot, with 94.0% deriving from five of them (croaker 35.2%, golden thread 23.6%, mackerel 15.2%, lizard fish 12.8% and toothed croaker 7.2%).

*Experimental design*

We received 221 Wistar-Kyoto rats (111 males, 110 females) aged 21 days on 6 June 1984 from Charles River Laboratories (Wilmington, MA, USA). Upon arrival, the rats were randomly assigned, within each sex, to one of three experimental groups. Rats in group 1 (37 males, 37 females) were fed a powder diet of one part salted fish to three parts certified rat chow (Certified Rodent Chow 5002, Ralston Purina Company) during the first 18 months. Similarly, rats in group 2 (37 males, 37 females) were fed a powder diet of one part salted fish to five parts certified rat chow during the first 18 months. Rats in group 3 (37 males, 36 females) were given rat chow only throughout the experiment. After the first 18 months, all rats were given rat chow pellets for the remaining time span of the experiment. The rats were housed in separate cages and weighed once a week during the first 78 weeks of the experiment. Thereafter, they were weighed once a month until age 3 years. All moribund rats or those that were alive at age 3 years (15 May 1987) were killed. A necropsy was performed on each rat by an experienced technician (J.E.) under the supervision of a pathologist (P.W.N.) and adhering to a standard protocol. Briefly, the rat was examined for external lesions and then laid on its back and pinned down. A midline incision was made from the mandibular symphysis to the anus. The skin was reflected so that mammary gland tissue, superficial lymph nodes and other subcutaneous structures could be examined. The abdomen and thorax were opened. Each organ system and regional lymph nodes were examined for abnormalities. All gross pathologic observations were recorded and all tumours were photographed. We excised and fixed in 10% buffered formalin tissues from all grossly visible tumours and gross lesions suspected of being tumours. Selected tumours were also fixed in gluteraldehyde. We routinely excised and fixed tissues from the lung, kidney, liver and stomach of each rat. The head of each rat was also routinely removed, fixed, decalcified and sagittal sections taken at different levels of the nasal and paranasal cavity. All sections were stained with Haematoxylin and Eosin for microscopic examination. Classification of tumours was according to Turusov (1973, 1976) and Jones *et al.* (1987).

*Statistical methods*

The life table method (Gart *et al.*, 1986) was employed to examine any difference in the overall survival or occurrence of specific tumours between the three experimental groups. The binomial test was used to compare the rate of nasal cavity cancer among rats treated with Chinese salted fish against the historical control incidence of this tumour in rats.

**Results and discussion**

The overall age at death was not statistically different between rats in the three experimental groups ($P = 0.28$). The respective median ages were: group 1 males, 130 weeks; group 1 females, 123 weeks; group 2 males, 131 weeks; group 2 females, 121 weeks; group 3 males, 129 weeks; and group 3 females, 127 weeks. At the start of the experiment, rats in the three experimental groups had similar body weights. Six months later, however, male rats in the high dose group started to show significantly lower weights relative to male rats in the control group, while male rats in the low dose group had intermediate weights. The same pattern was not evident among female rats until another 6 months later (i.e. 1 year after the start of the experiment). We terminated the experimental diets after 18 months, and almost immediately after that the treated rats began to gain weight such that at the second monthly weighing post-termination of the salted fish diets, no difference in weights was apparent between the three experimental groups. This pattern persisted until the end of the experiment.

**Table I** Occurrence of nasal cavity cancers in experimental animals

| *Diet group* | *No. rats* | *No. nasal cavity cancers* |
|---|---|---|
| Salted fish:rat chow (1:3) | 74 | 3 |
| Salted fish:rat chow (1:5) | 74 | 1 |
| Rat chow only | 73 | 0 |

**Figure 1** H and E section of the undifferentiated carcinoma showing a monomorphous population of cells with high nuclear cytoplasmic ratios and easily identifiable mitoses.

**Figure 2** H and E section of moderately differentiated squamous carcinoma showing nests of pleomorphic cells with focal keratinization.

There were four malignant nasal cavity tumours among rats fed the experimental diets (Table I). The first tumour (an undifferentiated carcinoma, Figure 1) was observed during week 44 of the experiment in a male rat belonging to the high dose group. The tumour largely replaced the mid and left lateral portions of the nasal cavity. It was composed of monomorphous sheets of primitive cells with high nuclear cytoplasmic ratios and a high mitotic rate. The second tumour (a moderately differentiated squamous cell carcinoma, Figure 2) was observed in a female rat, also belonging to the high dose group, during week 97 of the experiment. This tumour involved the left lateral nasal cavity midway between the eye and nares; it destroyed surrounding bony structures and extended into overlying soft tissues. Microscopic sections showed nests and cords of cells forming keratin pearls within a desmoplastic stroma. The third tumour (a spindle cell carcinoma, Figure 3) was observed in a second female rat of the high dose group during week 99 of the experiment. The tumour appeared to arise in the right posterior nasal cavity and to have extended along the auditory canal. Sections revealed that it enveloped epithelial structures presumed to be the eustachian tube and infiltrated adjacent soft tissues. The cells comprising the tumour have elliptical to spindled nuclei with moderate amounts of

**Figure 3** H and E section of spindled cell carcinoma showing a pleomorphic population of cells with a spindled appearance at the edge of the photomicrograph.

**Figure 4** H and E section of a spindled cell tumour showing a pleomorphic population of cells with elongated nuclei.

cytoplasm. Immunohistochemical stains showed the cells to have both keratin and vimentin in their cytoplasm. Electron microscopic studies revealed tight junctions, which supports the diagnosis. The fourth tumour (a spindle cell tumour not otherwise specified, Figure 4) was observed in a male rat of the low dose group during week 118 of the experiment. This tumour involved the left posterior nasal cavity and invaded adjacent soft tissues. It was composed of spindled cells with elongated nuclei. Immunohistochemical stains were inconclusive, showing the cells to be vimentin positive and keratin negative. Electron microscopic studies were performed and were also inconclusive. No tumours of the

**Table II** Tumours (other than nasal cavity) in experimental animals

| *Tumour* | *Salted fish: rat chow* 1:3 | 1:5 | *Rat chow only* |
|---|---|---|---|
| Endocrine | | | |
| Benign | 3 | 1 | 6 |
| Malignant | 0 | 1 | 1 |
| Mammary | | | |
| Benign | 9 | 7 | 7 |
| Malignant | 1 | 1 | 2 |
| Skin | | | |
| Benign | 4 | 3 | 1 |
| Malignant | 2 | 2 | 2 |
| Other | | | |
| Benign | 2 | 1 | 6 |
| Malignant | 7 | 4 | 10 |

upper respiratory tract were observed among control rats. The difference in the occurrence of these malignant tumours among the three experimental groups was marginally significant (one-sided $P$ for trend = 0.057). We also compared the rate of nasal cavity cancers among treated rats (4/148) against the historical control rate of zero (to our knowledge, there have been no reports of such spontaneous tumours in rats (Sher, 1982; Kroes *et al.*, 1981; Burek, 1978)), and the difference was statistically significant (one-sided $P = 0.02$). We observed no significant differences in the occurrence of any other tumours among the three experimental groups (Table II).

In order to relate the level of exposure in our rats to potential level of human exposure, one of the authors (M.C.Y.) interviewed 17 mothers in Guangzhou (a city in central Guangdong Province) to determine the amount of salted fish fed to young children who ate this food regularly. The 17 women were employees of the Sun Yat-Sen University of Medical Sciences, who had indicated during a screening interview that salted fish mixed with rice was a regular (at least five times a week) food for their children during and after weaning. Each woman was presented with a bucket of rice and pieces of salted fish in a separate bucket and asked to demonstrate the ratio of salted fish to rice when the two ingredients were mixed and fed to her children during and after weaning. She was asked to put the rice and salted fish separately in two small bowls; the contents of these two bowls were then weighed using a beam balance. For these 17 women, the ratio of salted fish to rice ranged from 1:18 to 1:6 with a median of 1:9. Therefore, the level of exposure to Chinese salted fish among our rats at the critical age (post-weaning) is quite close to that experienced by Chinese in Guangzhou who are at high risk of NPC. In Guangzhou, the lifetime risk (cumulative risk to age 65, the average life expectancy in China) of NPC for both sexes combined is 0.02 (Yu *et al.*, 1981). Our recently completed case–control study of NPC in Guangzhou indicated a relative risk of 2.1, and a population prevalence of 54% for exposure to salted fish during weaning (Yu *et al.*, 1989). We thus calculated that the lifetime risk of NPC in a Guangzhou resident exposed to salted fish post-weaning is 0.03. Among our rats fed one part salted fish to three parts rat chow, the lifetime risk of acquiring malignant tumour of the nasal cavity is 0.04 (3/74). A linear extrapolation from this rat model would then predict that the lifetime risk of NPC in an exposed resident of Guangzhou is 0.02. So, our rat model is in general agreement with the rate of NPC occurrence in a human population.

Low levels (sub-parts per million) of several volatile nitrosamines, including *N*-nitrosodimethylamine, *N*-nitrosodiethylamine, *N*-nitrosodi-*n*-propylamine, *N*-nitrosodi-*n*-butylamine and *N*-nitrosomorpholine, have been detected in samples of Chinese salted fish (Huang *et al.*, 1981; Tannenbaum *et al.*, 1985). Most of these volatile nitrosamines can induce nasal and paranasal cavity tumours in animals (Hass *et al.*, 1973; Pour *et al.*, 1973; Althoff *et al.*, 1974; Lijinsky & Taylor, 1978). In addition to these preformed nitrosamines, Tannenbaum *et al.* (1985) have detected bacterial mutagens in Chinese salted fish that had been exposed to a nitrosating agent under simulated gastric conditions. At present, it is not clear whether the volatile nitrosamines or bacterial mutagens present in Chinese salted fish are the putative carcinogens for NPC. The food may contain other types of carcinogenic substances which have not been identified; a systematic search for such substances is in progress.

In summary, epidemiological studies (Yu *et al.*, 1986, 1988, 1989) have shown a strong positive association between intake of Chinese salted fish early in life and subsequent development of NPC. In the present experiment, we observe a dose-dependent occurrence of malignant tumours of the nasal cavity among rats fed the same cooked food post-weaning, and in an amount resembling the level of potential human exposure at the corresponding young age. Our results, therefore, further strengthen the hypothesis that Chinese salted fish is a human nasopharyngeal carcinogen; they also establish Wistar rats as a viable animal model for carcinogenicity studies of this food in the laboratory.

This work was supported by United States Public Health Service grants KO4CA00884 and RO1CA40468 from the National Cancer Institute, and grant SIG-2 from the American Cancer Society. The authors thank Qing-Sheng Wang for his help during the critical early phase of the experiment, Shiu-Hung Lai for purchasing the salted fish used in the experiment and Kazuko Arakawa for her assistance in data analysis.

### References

ALTHOFF, J., MOHR, U., PAGE, N. & REZNICK, G.J. (1974). Carcinogenic effect of dibutylnitrosamine in European hamsters (*Cricetus cricetus*). *J. Natl Cancer Inst.*, **53**, 795.

BOYD, E.M. (1973). *Toxicity of Pure Foods*. CRC Press: Cleveland.

BUREK, J.D. (1978). *Pathology of Aging Rats*. CRC Press: West Palm Beach.

CHARLES RIVER TECHNICAL BULLETIN (1982). Volume 1, no. 1, The Charles River Laboratories Inc., Wilmington, MA.

GART, J.J., KREWSKI, D., LEE, P.N., TARONE. R.E. & WAHRENDORF, J. (1986). *Statistical Methods in Cancer Research. Volume III, the Design and Analysis of Long-term Animal Experiments*, IARC Sci. Publ. no. 79. International Agency for Research on Cancer: Lyon.

HASS, H., MOHR, U. & KRUGER, F.W. (1973). Comparative studies with different doses of *N*-nitrosomorpholine, *N*-nitrosopiperidine, *N*-nitrosomethylurea and dimethylnitrosamine in Syrian golden hamsters. *J. Natl Cancer Inst.*, **51**, 1295.

HO, J.H.C. (1971). Genetic and environmental factors in nasopharyngeal carcinoma. In *Recent Advances in Human Tumor Virology and Immunology*, Nakahara, W., Nishioka, K., Hitayama, T. & Ito, Y. (eds) p. 275. Tokyo University Press: Tokyo.

HUANG, D.P., HO, J.H.C., SAW, D. & TEOH, T.B. (1978). Carcinoma of the nasal and paranasal regions in rats fed Cantonese salted marine fish. In *Nasopharyngeal Carcinoma: Etiology and Control*, De-The, G. & Ito, Y. (eds) p. 315. IARC: Lyon.

HUANG, D.P., HO, J.H.C., WEBB, K.S., WOOD, B.J. & GOUGH, T.A. (1981). Volatile nitrosamines in salt-preserved fish before and after cooking. *Fd Cosmet. Toxicol.*, **19**, 167.

JONES, T.C., MOHR, U. & HUNT, R.D. (1987). *Genital System*. Monographs on Pathology of Laboratory Animals. Springer-Verlag: Berlin.

KROES, R., GARBIS-BERKVENS, J.M., DE VRIES, T. & NESSELROOY, J.H.J. (1981). Histopathological profile of a Wistar rat stock including a survey of the literature. *J. Gerontol.*, **36**, 259.

LIJINSKY, W. & TAYLOR, H.W. (1978). Relative carcinogenic effectiveness of derivatives of nitrosodiethylamine in rats. *Cancer Res.*, **38**, 2391.

McCARTHY, J.P. & TAUSZ, J. (1952). *Salted Fish Industry in Hong Kong*. Government Printer: Hong Kong.

NATIONAL CANCER CONTROL OFFICE, NANJING INSTITUTE OF GEOGRAPHY (1980). *Atlas of Cancer Mortality in the People's Republic of China*. China Map Press: Shanghai.

POUR, P., KRUGER, F.W., CARDESA, A., ALTHOFF, J. & MOHR, U. (1973). Carcinogenic effect of di-*n*-propylnitrosamine in Syrian golden hamsters. *J. Natl Cancer Inst.*, **51**, 1019.

SHER, S.P. (1982). Tumors in control hamsters, rats, and mice: literature tabulation. *CRC Crit. Rev. Toxicol.*, **10**, 49.

TANNENBAUM, S.R., BISHOP, W., YU, M.C. & HENDERSON, B.E. (1985). Attempts to isolate *N*-nitroso compounds from Chinese-style salted fish. *Natl Cancer Inst. Monogr.*, **69**, 209.

TURUSOV, V.S. (1973). *Pathology of Tumors in Laboratory Animals, Volume 1, Tumors of the Rat. Part 1*. IARC Sci. Publ. no. 5. International Agency for Research on Cancer: Lyon.

TURUSOV, V.S. (1976).*Pathology of Tumors in Laboratory Animals, Volume 1, Tumors of the Rat. Part 2*. IARC Sci. Publ. no. 6. International Agency for Research on Cancer: Lyon.

YU, M.C., HO, J.H.C., LAI, S.H. & HENDERSON, B.E. (1986). Cantonese-style salted fish as a cause of nasopharyngeal carcinoma: report of a case–control study in Hong Kong. *Cancer Res.*, **46**, 956.

YU, M.C., HO, J.H.C., ROSS, R.K. & HENDERSON, B.E. (1981). Nasopharyngeal carcinoma in Chinese – salted fish or inhaled smoke. *Prev. Med.*, **10**, 15.

YU, M.C., HUANG, T.-B. & HENDERSON, B.E. (1989). Diet and nasopharyngeal carcinoma: a case–control study in Guangzhou, China. *Int. J. Cancer* (in the press).

YU, M.C., MO, C.-C., CHONG, W.-X., YEH, F.-S. & HENDERSON, B.E. (1988). Preserved foods and nasopharyngeal carcinoma: a case–control study in Guangxi, China. *Cancer Res.*, **48**, 1954.

WATERHOUSE, J., SHANMUGARATNAM, K., MUIR, C. & POWELL, J. (1982). *Cancer Incidence in Five Continents, Vol. 4*. IARC Sci. Publ. no. 42. International Agency for Research on Cancer: Lyon.